# *Pseudomonadota* in the oral cavity: a glimpse into the environment-human nexus

**DOI:** 10.1007/s00253-022-12333-y

**Published:** 2022-12-26

**Authors:** Inês Leão, Teresa Bento de Carvalho, Valentina Henriques, Catarina Ferreira, Benedita Sampaio-Maia, Célia M. Manaia

**Affiliations:** 1 Universidade Católica Portuguesa, CBQF - Centro de Biotecnologia e Química Fina – Laboratório Associado, Escola Superior de Biotecnologia, Porto, Portugal; 2 i3S-Instituto de Investigação e Inovação em Saúde, Universidade do Porto, Porto, Portugal; 3 Faculdade de Medicina Dentária da Universidade do Porto, Porto, Portugal

**Keywords:** Human–environment nexus, Health, Saliva, Virulence factors, Antibiotic resistance, Ubiquity, One Health

## Abstract

**Abstract:**

The phylum *Pseudomonadota* is amongst the most represented in the environment, with a comparatively lower prevalence in the human oral cavity. The ubiquity of *Pseudomonadota* and the fact that the oral cavity is the most likely entry portal of bacteria from external sources underlie the need to better understand its occurrence in the interface environment-humans. Yet, the relevance oral *Pseudomonadota* is largely underexplored in the scientific literature, a gap that this review aims at addressing by making, for the first time, an overview of the diversity and ecology of *Pseudomonadota* in the oral cavity. The screening of scientific literature and human microbiome databases unveiled 1328 reports of *Pseudomonadota* in the oral cavity. Most of these belonged to the classes *Beta-* and *Gammaproteobacteria*, mainly to the families *Neisseriaceae*, *Campylobacteriaceae*, and *Pasteurelaceae*. Others also regularly reported include genera such as *Enterobacter*, *Klebsiella*, *Acinetobacter*, *Escherichia*, *Burkholderia*, or *Citrobacter*, whose members have high potential to acquire virulence and antibiotic resistance genes. This review provides evidence that clinically relevant environmental *Pseudomonadota* may colonize humans via oral cavity. The need for further investigation about *Pseudomonadota* at the environment-oral cavity interface and their role as vectors potentially involved in virulence and antibiotic resistance transmission is demonstrated.

**Key points:**

• *Neisseriaceae, Campylobacteriaceae, and Pasteurelaceae are part of the core oral microbiome*

• *Enterobacteriaceae, Acinetobacter, or Burkholderia are frequent in the oral microbiome*

• *Gut dysbiosis may be associated with colonization by ubiquitous oral Pseudomonadota*

**Graphical abstract:**

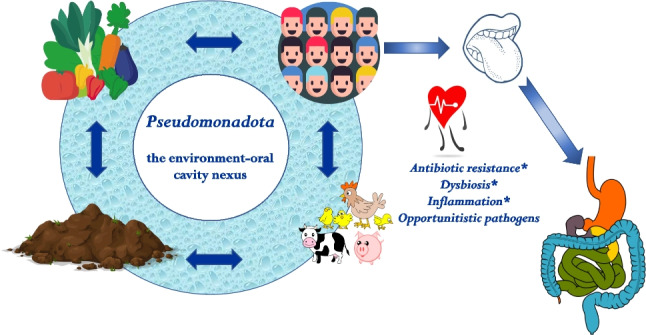

**Supplementary information:**

The online version contains supplementary material available at 10.1007/s00253-022-12333-y.

## Introduction

The human microbiome is a key player in the balance between health and disease. Insights into the diversity and organization of the complex microbial ecosystem that inhabits the human oral cavity are crucial to understand possible impacts on health and disease, at the oral or systemic levels (Wade 2013; Tuganbaev et al. 2022). Oral microbiomes are characterized by high richness and diversity. About 800 bacterial species have been reported in the human mouth and aerodigestive tract (i.e. pharynx, nasal passages, sinuses, and esophagus), most of which (76%) are culturable, although only 58% are officially named (The Human Microbiome Oral Database, http://www.homd.org – HOMD V3, accessed at 23 November 2022). More than half of these taxa (481 out of 789) are specifically associated with a nasal/oral or oral body site and observed to belong to 12 *Bacteria* and one *Archaea* phyla (HOMD V3).

In healthy humans, the core oral microbiome is dominated by members of six phyla, which account for more than 90% of the taxa identified (HOMD V3): *Bacillota* (formerly *Firmicutes*), *Actinobacteriota* (*Actinobacteria*), *Pseudomonadota* (formerly *Proteobacteria*), *Fusobacteriota* (formerly *Fusobacteria*), *Bacteroidota* (formerly *Bacteroidetes*), and *Spirochaetota* (formerly *Spirochaetes*) (Deo and Deshmukh 2019; Zhang et al. 2018). Nonetheless, the structure of the core microbiota, i.e. the relative proportions of each taxon, is supposed to vary. Determinant factors include the geography and diet, the anatomic and physiologic characteristics of the host, as well as the oral cavity site, and consequent biofilm formation, oxygen and nutrient availability, exposure to host immunological factors, among others (Zhigang Ren et al. 2021; Sampaio-Maia et al. 2016; Zhang et al. 2018; Wang et al. 2022b). The broad diversity of oral microbiomes was evidenced by Tierney et al. (2019) who, based on the analysis of 1473 oral metagenomes, identified 23 961 508 genes, half of which were unique in each metagenome. Such a uniqueness has led some authors to propose the oral microbiome profile as a valuable tool for biogeography investigations or forensic personal discrimination (Wang et al. 2022a, 2022b).

The interconnections between phylogenetic diversity and ecology of the microbiota inhabiting the oral cavity, and the possible relationship with host conditions (e.g. age, diet, physical condition), are supposed to have important implications in human health (Burcham et al. 2020; Sampaio-Maia et al. 2016; Tang et al. 2019; Hayes et al. 2018; Dashper et al. 2019). Indeed, the nexus between the oral and gut microbiome is a topic of interest in the exploitation of the oral microbiota (Teil Espina et al. 2019; Iwauchi et al. 2019). Considering that a milliliter of saliva may contain 8–9 log-units of microbial cells, some of which can multiply every 3–4 h, it can be estimated that 1–3 g of microbial biomass can be ingested per day (Edgar et al. 2012). Given the capacity of some of these microbial cells to survive and colonize the host’s gut, a balanced and stable oral microbiota is an essential barrier to prevent pathogen colonization and infection and, therefore, oral and/or systemic infections and/or inflammatory symptoms (Albuquerque-Souza and Sahingur 2022; Ren et al. 2021; Sampaio-Maia et al. 2016; Willis et al. 2020). The interface environment-human has received a renewed attention under the One Health perspective that considers a continuum between humans, animals, and the natural environment (Cunningham et al. 2017; Osterhaus et al. 2020). Accordingly, the human microbiome is affected by the surrounding environment, with the geography, diet, and lifestyle shaping its structure (Cunningham et al. 2017; Osterhaus et al. 2020). In particular, the oral microbiome is expected to be influenced by external conditions that include not only the range of microorganisms to which humans are exposed, for instance, via food products, but also by lifestyle and hygiene habits (Freire et al. 2020; Peters et al. 2018; Tang et al. 2019; Wang et al. 2022a, 2022b).

*Pseudomonadota* are reported among the predominant phyla in the natural environments, which frequently are under anthropic impacts (Chen et al. 2019; Ferro et al. 2019; Higgins et al. 2018; Nazareno Scaccia et al. 2021; Vaz-Moreira et al. 2014; 2017). In turn, *Pseudomonadota* include some of the most ubiquitous bacterial groups, as well as a vast array of opportunistic pathogens, for which existing antibacterial drugs may be ineffective (e.g. *Acinetobacter*, *Pseudomonas*, *Enterobacteriaceae*) (Ferro et al. 2019; Jordi Rello et al. 2019; Vaz-Moreira et al. 2014; Rizzatti et al. 2017; Theuretzbacher et al. 2020). Curiously, in spite of these clinically relevant features and although *Pseudomonadota* are the second most abundant phylum in the mouth (Wang et al. 2022b), not much attention has been given to its presence. Indeed, in discussions about the oral microbiome and its relevance, *Pseudomonadota* are frequently underexplored when compared with other groups (Radaic and Kapila 2021; Zhang et al. 2018). This review aims at addressing this topic by making, for the first time, an overview of the diversity and ecology of *Pseudomonadota* in the oral cavity.

In healthy individuals, oral *Pseudomonadota* are mostly represented by members of the families *Neisseriaceae*, *Pasteulleraceae*, and *Campylobacteraceae*, although the distribution of bacteria may be site- and subject-specific (Aas et al. 2005; Jiang et al. 2019; Zaura et al. 2009). It has been also reported that *Pseudomonadota* tend to increase in the oral cavity with the age and are frequently associated with inflammatory diseases (Iwauchi et al. 2019; Singh et al. 2019) and other non-infectious disorders (Costa et al. 2021; Rizzatti et al. 2017). However, further insights about *Pseudomonadota* diversity in the oral cavity will better elucidate the role of the oral cavity as an entry portal for environmental clinically relevant bacteria. With the increasing capacity to have holistic insights across the One Health microbiomes, new opportunities to explore the environment-oral-gut microbiome nexus emerge. This perspective not only will shed additional light into beneficial interactions and how they can promote health, but also will contribute to better understand how some antibiotic resistance or virulence determinants may have access into the human oral microbiome, or how the oral microbiome may be related with health impairment.

This review addresses the hypothesis that the human oral cavity is exposed to different groups of *Pseudomonadota*, some of which can also thrive in the external environment. It is also hypothesized that given their specific physiological and biochemical, intrinsic or acquired, properties some of these *Pseudomonadota* may be able to colonize other parts of the human body, and act as vectors of antibiotic resistance or as opportunistic infections. The approach to test the abovementioned hypotheses was to review the literature and databases available, list the most commonly reported *Pseudomonadota* in the oral cavity and, based on this information, infer about their ecology and distribution, presence of potential clinically relevant properties, and discuss possible implications for the human oral microbiome.

## Oral *Pseudomonadota* in the scientific literature and in public databases

A total of eighty-seven publications surveying the microbiota in the human oral cavity, and reporting target populations, samples’ characteristics, and identification methods (Tables S1 and S2), were screened for the presence of *Pseudomonadota*. In addition, *Pseudomonadota* taxa reports in the oral cavity were searched in the databases NIH Human Microbiome Project (https://www.hmpdacc.org/) (*n* = 24) and expanded Human Oral Microbiome Database (http://www.homd.org/) (*n* = 53) (Table S1 and S2). The bacterial groups were listed and categorized according to the number of reports (number of times that a taxonomic group was identified) and frequency (quotient between the total number of identifications of a specific taxonomic group and the total number of taxa reported) (Table S3). The data was organized at the genus level, meaning that each name referred to the sum of all taxa within that genus, regardless the identification to the species level. In studies based on the 16S rRNA gene amplicon sequencing, the absence/presence of a given taxon was considered, disregarding relative abundance values.

Genera that were reported more than five times were characterized for their environmental distribution, and possible carriage of antibiotic resistance and/or virulence determinants. Common habitats were compiled based on the section of ecology and habitats of Bergey’s Manual (Garrity et al. 2005) and Nørskov-Lauritsen and Kilian (2006) for the genus *Aggregatibacter*. Antibiotic resistance and virulence genetic determinants were searched based on whole genome sequences available in the Pathosystems Resource Integration Center (PATRIC, https://www.patricbrc.org, accessed January 2021), through the Specialty Genes tab—The Antibiotic Resistance Database (ARDB) (Liu and Pop 2009) and the Comprehensive Antibiotic Resistance Database (CARD) (McArthur et al. 2013), and Victors virulence factors database (Sayers et al. 2019) and the Virulence Factor Database (VFDB) (Chen et al. 2016), respectively. The results were filtered for 100% sequence identity and subject/query coverage, and duplicates were deleted. The genes or gene-product designations were browsed in CARD and VFDB databases for verification of the antibiotic resistance and virulence mechanisms, respectively. The phylogenetic relationship between taxa reported in the oral cavity and human gut microbiota was assessed based on the 16S rRNA gene sequences available for the type strains of the species (https://lpsn.dsmz.de; Parte et al. 2020), used as query for mega BLAST search using the filter human gut microbiome containing 9759 16S ribosomal RNA gene sequences (https://blast.ncbi.nlm.nih.gov/Blast.cgi; (Zhang et al. 2000) (accessed at 7th May 2022).

## *Pseudomonadota* in the oral cavity

The literature reviewed was published between 2005 and 2020 and relied on 16S rRNA gene amplicon sequencing (*n* = 76), mainly based on Illumina or 454 pyrosequencing, and on culture-based methods (*n* = 6) or targeted methods based on PCR or probe hybridization (*n* = 7) (Table S1). Most of the studies used samples of saliva or mouth washes, tooth or dental plaques, or soft tissues such as gingiva, cheek, tongue, or tonsils (Table S2). The number of individuals analysed per study ranged between 2 and 2338, with ages between 18 and 80 years, although the information about the studied group was not clearly provided in 12 publications. Some studies included individuals with specific conditions, such as cancer patients (6 articles) or smoker groups (6 articles), or an indigenous tribe (1 article). Based on this search, it was possible to list 1328 observations that corresponded to a total of 313 genera, of which 77 were reported three or more times (i.e. *n* = 27 three times, *n* = 10 four times, and *n* = 40 five times or more) (Table S2, S3). The remaining were reported only once (*n* = 191) or twice (*n* = 45) (Table S3a). The fact that distinct analytical and identification methods were used in different studies might have led to distinct identifications at the species level, a bias that we believe was considerably reduced at the genus or higher taxonomic ranks, as we used in this study (Table S3).

**Fig. 1:**
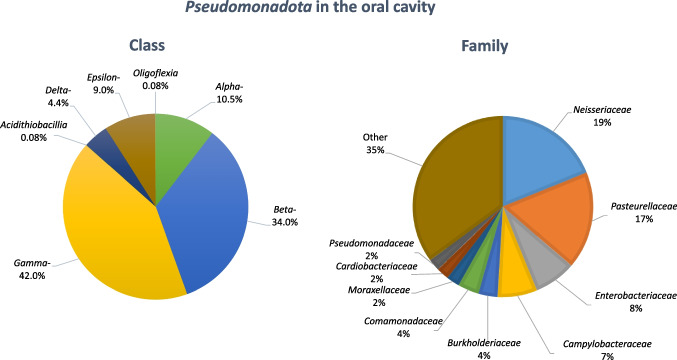
*Pseudomonadota* in the oral cavity number of reports identified at the class or family levels

Most of the 1328 observations of *Pseudomonadota* in the oral cavity (Fig. 1; Table S3a) belonged to the classes *Gammaproteobacteria* (*n* = 558) and *Betaproteobacteria* (*n* = 452). Members of the classes *Alphaproteobacteria* (*n* = 139), *Epsilonproteobacteria* (*n* = 119), and *Deltaproteobacteria* (*n* = 57) were reported fewer times, and the classes *Acidithiobacillia* and *Oligoflexia* were mentioned only three times. More than half of the 1328 observations were included in four families, specifically *Neisseriaceae* (*n* = 263, class *Betaproteobacteria*), represented by 10 genera, *Pasteurellaceae* (*n* = 243, class *Gammaproteobacteria*) distributed by 13 genera, *Enterobacteriaceae* (*n* = 100, class *Gammaproteobacteria*) that included 12 genera, and *Campylobacteraceae* (*n* = 99, class *Epsilonproteobacteria*) that although represented by two genera, most (*n* = 98) referred to the genus *Campylobacter* (Table S3a). These observations agree with previous studies that refer to *Neisseriaceae*, *Pasteurellaceae*, and *Campylobacteraceae* as the most common *Pseudomonadota* of the oral microbiome (Zaura et al. 2009). However, among the genera that were reported five times or more (Table S3c) (*n* = 926), it noteworthy the occurrence of other *Pseudomonadota*, such as members of the families *Enterobacteriaceae* (*n* = 48, 5%), *Burkholderiaceae* (*n* = 43; 5%), *Comamonadaceae* (*n* = 9, 1%), *Moraxellaceae* (*n* = 31, 3%), and *Pseudomonadaceae* (*n* = 29, 3%) that include bacteria with potential clinical relevance, recurrently reported in the oral cavity (Table S3c, Table 1). For example, members of the family Enterobacteriaceae were reported in 25 distinct publications (Table S2, S3). The occurrence of taxa that do not belong to the core oral microbiome may be favoured in some conditions. For example, a recent study suggested that when compared to healthy controls, chronic kidney disease patients presented the proliferation of clinically relevant *Enterobacteriaceae* in the oral cavity, potentially harbouring acquired antibiotic resistance genes (Costa et al. 2021). Another example was provided by a mice model study, which showed that periodontitis was associated with the accumulation in the oral cavity of *Enterobacteriaceae*, specifically of the genera *Klebsiella* and *Enterobacter* (Kitamoto et al. 2020).

**Table 1:** Diversity and characteristics of the genera of bacteria belonging to the phylum *Pseudomonadota*, whose occurrence in the oral cavity was reported more than five times in the examined literature. In a total of 1328 genera of *Pseudomonadota*, 583 were reported more than five times and were affiliated to (class, number of genera, number of observations) the following: *Alphaproteobacteria*, 4, 27; *Betaproteobacteria*, 11, 327; *Deltaproteobacteria*, 3, 24; *Epsilonproteobacteria*, 2, 109; *Gammaproteobacteria* 20, 439. Virulence and antibiotic resistance characteristics of members of these genera were collected from the public database PATRIC (https://www.patricbrc.org/) and the following sources: ARDB, CARD, Victors, VFDB. Ecology and habitats were reviewed from the Bergey’s Manual (Garrity et al. 2005) and (Nørskov-Lauritsen and Kilian 2006) for *Aggregatibacter*. Additional information is provided as a supplementary file

Reported diversity in the oral cavity			Ecology and habitats	Genome-based characterization of genus members			
Top	Genus Class Family Nº reports-genus/total	Reported species (nº)	Common habitats	Virulence^1^	Other taxa (%)^2^	Antibiotic resistance^1^	Other taxa (%)^2^
1	*Neisseria* *Neisseriaceae* *Beta-* 165/1328	*N. subflava* (17) *N. flavescens* (8) *N. mucosa* (7) *N. elongata* (7) *N. sicca* (5) *N. bacilliformis* (5) *N. weaveri* (4) *N. oralis* (4) *N. lactamica* (4) *N. flava* (4) *N. polysaccharea* (3) *N. pharyngis* (3) *N. meningitidis* (3) *N. perflava* (2) *N. gonorrhoeae* (2) *N. cinerea* (2) *N. dentiae* (1) *N. canis* (1) *N. animalis* (1) *Neisseria* spp*.* (82)	Mucous membrane surface (oropharynx, nasopharynx, throat)	Adherence Invasion Motility Transport Iron transport Protease Stress proteins Protease Host response evasion Endotoxin	*Neisseria* 100*%*	Target modification Efflux	*Neisseria* 100*%*
2	*Haemophilus* *Pasteurellaceae* *Gamma-* 110/1328	*H. parainfluenzae* (16) *H. haemolyticus* (5) *H. paraphrohaemolyticus* (4) *H. sputorum* (3) *H. pittmaniae* (3) *H. influenzae* (3) *H. aegyptius* (3) *H. parahaemolyticus* (2) *H. haemoglobinophilus (*1) *H. ducreyi* (1) *Haemophilus* spp*.* (69)	Mucous membranes surface (upper respiratory tract, oral cavity)	Invasion Iron transport Transport Protease Host response evasion Endotoxin Others	*Haemophilus* 96.7%	Efflux Inactivation Target modification Other	*Salmonella* 62.7% *Serratia* 15.4% *Haemophilus* 7.5% *Mannheimia* 6.0%
3	*Campylobacter* *Campylobacteraceae* *Epsilon* 98/1328	*C. gracilis* (10) *C. concisus* (10) *C. showae* (9) *C. rectus* (7) *C. curvus* (4) *C. sputorum* (2) *C. lari (*1) *C. jejuni* (1) *C. insulaenigrae* (1) *C. hyointestinalis*(1) *C. hominis* (1) *C. helveticus* (1) *C. fetus* (1) *C. ureolyticus* (1) *Campylobacter* spp*.* (48)	Reproductive organs, intestinal tract and oral cavity (humans and animals)	Adherence Invasion; Motility/Chemotaxis Host response evasion Secretion system; Others	*Campylobacter* 100%	Efflux Inactivation Target modification	*Campylobacter* 98.7%
4	*Aggregatibacter* *Pasteurellaceae* *Gamma-* 85/1328	*A. actinomycetemcomitans* (9) *A. segnis* (9) *A. aphrophilus* (6) *A. paraphrophilus* (5) *Aggregatibacter* spp*.* (56)	Dental surfaces Pharynx, also peritoneum, pleura and bone	None	None	None	None
5	*Kingella* *Neisseriaceae* *Beta-* *53*/1328	*K. denitrificans* (6) *K. kingae* (3) *K. oralis*(6) *Kingella* spp.*(*38*)*	Mucous membranes, upper respiratory tract and oral cavity, also urogenital (humans and other primates)	None	None	Efflux Inactivation Target modification	*Neisseria 22.2%* *Morganella 22.2%* *Vibrio 22.2%* *Salmonella 22.2%* *Serratia 11.1%*
6	*Cardiobacterium* *Cardiobacteriaceae* *Gamma-* 30/1328	*Card. hominis* (6) *Card. valvarum* (5) *Cardiobacterium* spp*.* (19)	Mucous membranes (nose, mouth, and throat and also gastrointestinal tract)	None	None	None	None
	*Lautropia* *Burkholderiaceae Beta-* 30/1328	*L. mirabilis* (10) *Lautropia* spp*.* (20)	Oral cavity (gingival surface)	None	None	None	None
7	*Pseudomonas* *Pseudomonadaceae* *Gamma-* 29/1328	*P. aeruginosa* (6) *P. fluorescens* (3*)* *P. stutzeri* (2) *P. beteli(*1) *P. fragi* (1) *P. luteola* (1*)* *P. otitidis* (1) *P. pseudoalcaligenes* (1) *Pseudomonas* spp*.* (13)	Natural environments (water, soil, plants), and animal tissues. Include human pathogens	Adherence Invasion Motility/Chemotaxis Transport Iron transport Protease Stress proteins Quorum sensing Protease Lipase Host response evasion Secretion system Endotoxin Toxins	*Pseudomonas* 100%	Efflux Inactivation Target modification Reduced permeability	*Pseudomonas* 92.7%
8	*Eikenella* *Neisseriaceae* *Beta-* 27/1328	*E. corrodens* (9) *Eikenella* spp. (18)	Upper respiratory tract, oral cavity and subgingival plaque (periodontitis)	None	None	None	None
9	*Actinobacillus* *Pasteurellaceae* *Gamma-* 23/1328	*Act. capsulatus* (1) *Act. equuli* (1) *Act. minor* (1) *Act. pleuropneumoniae* (1) *Act. porcinus* (1) *Act. scotiae* (1) *Act. succinogenes* (1) *Act. ureae* (1) *Actinobacillus* spp. (15)	Mucous membranes of different carrier animals (humans, sheep, cattle, horses, pigs, other mammals and birds)	Motility Transport Iron transport Others	*Actinobacillus* 99.9%	Efflux Inactivation Target modification	*Vibrio* 41.2% *Serratia* 22.1% *Mannheimia* 17.6% *Escherichia* 12.2%
	*Enterobacter* *Enterobacteriaceae* *Gamma-* 23/1328	*Ent. cloacae (*4) *Ent. aerogenes* (3) *Ent. sakazakii* (3) *Ent. gergoviae* (2) *Ent. hormaechei* (2) *Ent. amnigenus* (1) *Ent. cancerogenus* (1) *Ent. mori* (1) *Ent. xiangfangensis* (1) *Enterobacter* spp*.*(5)	Natural environments (water, soil, plants), and animal tissues. Include human pathogens	Adherence Invasion Transport Iron transport Motility/Chemotaxis Secretion system; Toxins Lipase Host response evasion Other	*Salmonella* 49.0% *Escherichia* 24.8% *Shigella* 20.9%	Efflux Inactivation Target modification Other	*Klebsiella* 21.2% *Vibrio* 18.1% *Enterobacter* 13.8% *Salmonella* 13.0% *Escherichia* 13.1% *Pseudomonas* 7.0%
10	*Klebsiella* *Enterobacteriaceae* *Gamma-* 23/1328	*Kl. pneumoniae* (10) *Kl. oxytoca* (4) *Kl. singaporensis* (1) *Kl. michiganensis* (1) *Klebsiella* spp. (7)	Natural environments (water, soil, plants), and animal tissues Include human pathogens	Transport Iron transport Others	*Salmonella* 33.7% *Escherichia* 32.8% *Shigella* 31.7%	Efflux Inactivation Target modification Reduced permeability Other	*Klebsiella* 43.1% *Escherichia* 15.7% *Vibrio* 12.6% *Enterobacter* 7.9% *Salmonella* 6.5%
	*Serratia* *Yersiniaceae* *Gamma-* 17/1328	*S. marcescens* (6) *S. ficaria* (2) *S. liquefaciens* (1) *S. rubidaea* (1) *S. odorifera* (1) *Serratia* spp. (6)	Natural environments (water, soil, plants), and animal tssues. Include human pathogens	Transport Iron transport Others	*Yersinia* 98.6%	Efflux Inactivation Target modification Other	*Klebsiella* 25.2% *Escherichia* 15.6% *Salmonella* 10.6% *Serratia* 23.1% *Vibrio* 11.7%
11	*Acinetobacter* *Moraxellaceae* *Gamma-* 16/1328	*Ac.baumannii (*5) *Ac. johnsonii (*1) *Ac. schindleri* (1) *Ac. junii*(1) *Acinetobacter* spp*.* (8)	Natural environments (water, soil, plants), and animal tissues. Include human pathogens	Adherence Invasion Transport Iron transport Secretion systems Motility/Chemotaxis Toxins Host response evasion Others	*Pseudomonas* 75.0% *Staphylococcus* 13.5%	Efflux Inactivation Target modification Other	*Acinetobacter* 76.3% *Vibrio* 5.9%
12	*Escherichia* *Enterobacteriaceae* *Gamma-* 12/1328	*Esch. coli* (4) *Escherichia* spp. (8)	Gastrointestinal tract of warm-blooded animals. Also in natural environments (water, soil, plants)	Adherence Invasion Transport Iron transport Protease Lipase Secretion systems Motility/Chemotaxis Toxins Host response evasion Others	*Escherichia* 69.8% *Shigella* 25.0%	Efflux Inactivation Target modification Reduced permeability Other	*Escherichia* 97.0%
13	*Helicobacter* *Helicobacteraceae* *Epsilon-* 11/1328	*H. pylori* (7) *H. trogontum* (1) *Helicobacter* spp. (3)	Gastrointestinal tract, oral cavity and internal organs of humans and animals	Adherence Motility Endotoxin Toxin Secretion systems Acid resistance Others	*Helicobacter* 100%	Efflux Inactivation Other	*Helicobacter* 86.1% *Campylobacter* 9.1%
	*Simonsiella* *Neisseriaceae* *Beta-* 11/1328	*Sim. muelleri (*6) *Simonsiella* spp. (5)	Mucous membranes, oral cavity of warm-blooded animals	None	None	None	None
	*Sphingomonas* *Sphingomonadaceae* *Alpha-* 11/1328	*Sph. yabuuchiae* (2) *Sphingomonas* spp. (9)	Natural environments (water, soil, plants)	Adherence Motility Iron transport Secretion systems Host response evasion Protease Others	*Pseudomonas* 86.3% *Streptococcus* 13.7%	Efflux Inactivation Target modification	*Pseudomonas* 90.5% *Vibrio* 7.1%
14	*Desulfobulbus* *Desulfobulbaceae* *Delta-* 10/1328	*D. elongates* (1) *D. mediterraneus (*1) *D. rhabdoformis* (1) *Desulfobulbus* spp*.* (7)	Natural environments (anoxic black mud, sewage, fresh- or brackish water) and gastrointestinal tract of animals	None	None	None	None
	*Moraxella* *Moraxellaceae* *Gamma-* 10/1328	*M. oblonga* (1) *M. osloensis* (1) *M. catarrhalis* (1) *Moraxella* spp. 7/10	Mucous membranes, oral cavity of warm-blooded animals	Adherence Motility	*Helicobacter* 100%	Efflux Inactivation Target modification	*Vibrio* 27.3% *Escherichia* 27.3% *Mannheimia* 9.1% *Pasteurella* 18.2% *Psychrobacter* 9.1% Others 9.1%
15	*Comamonas* *Comamonadaceae* *Beta-* 9/1328	*Com. testosteroni* (2) *Com. terrigena* (1) *Comamonas* spp. (6)	Natural environments (water, soil, plants) Polluted environments	None	None	Efflux Inactivation Target modification	*Vibrio* 28.6% *Escherichia* 28.6% *Acinetobacter* 10.7% *Enterobacter* 10.7% *Pseudomonas* 10.7% *Aeromonas* 7.1%
16	*Aeromonas* *Aeromonadaceae* *Gamma-* 8/1328	*Aero. hydrophila* (1) *Aero. veronii* (1) *Aeromonas* spp. 6/8	Natural environments (fresh- or brackish-water, sewage)	Adherence Secretion systems Others	*Aeromonas* 99.0%	Efflux Inactivation Target modification Other	*Vibrio* 24.7% *Aeromonas* 13.7% *Klebsiella* 13.3% *Escherichia* 10.1% *Acinetobacter* 7.1% *Salmonella* 7.8%
	*Burkholderia* *Burkholderiaceae* *Beta-* 7/1328	*B. cepacia* (1) *Burkholderia* spp. (6)	Natural environments (water, soil, plants), and animal tissues. Include human pathogens	Adherence Invasion Motility/Chemotaxis Secretion system Host response evasion Others	*Burkholderia* 100%	Efflux Inactivation Target modification Other	*Burkholderia* 81.5% *Acinetobacter* 5.9% *Pseudomonas* 5.9%
	*Citrobacter* *Enterobacteriaceae* *Gamma-* 8/1328	*Cit. koseri* (2) *Cit. amalonaticus* (1) *Cit. freundii* (1) *Citrobacter* spp. (4)	Gastrointestinal tract of warm-blooded animals. Also in natural environments (water, soil, plants)	Iron transport Others	*Salmonella* 54.6% *Escherichia* 21.7% *Shigella* 21.4%	Efflux Inactivation Target modification Other	*Escherichia* 19.8% *Vibrio* 15.2% *Klebsiella* 14.2% *Citrobacter* 11.6% *Enterobacter* 7.7% *Salmonella* 9.3% Others 5.8%
	*Herbaspirillum* *Oxalobacteraceae* *Beta-* 8/1328	*Herb. hiltneri* (1) *Herb. frisingense* (1) *Herb. chlorophenolicum* (1) *Herb. seropedicae* (1) *Herb. lusitanum* (1) *Herb. rubrisubalbicans* (1) *Herb. huttiense* (1) *Herbaspirillum* sp. (1)	Plants (gramineous; roots, stems and leaves)	None	None	Inactivation	*Escherichia 100%*
	*Pasteurella* *Pasteurellaceae* *Gamma-* 8/1328	*Past. pneumotropica* (1) *Past. multocida* (1) *Past. mairii* (1) *Pasteurella* spp. (5)	Mucous membranes (upper respiratory tract) and lower genital tracts of mammals (rarely humans) and birds	Adherence Motility Others	*Pasteurella* 100%	Efflux Inactivation Target modification	*Vibrio* 21.9% *Serratia* 32.5% *Escherichia* 7.5% *Manheimia* 6.3% *Pasteurella* 10.6% *Pseudomonas* 5.0% Others 5.6%
	*Stenotrophomonas* *Lysobacteraceae* *Gamma-* 8/1328	*Sten. maltophilia* (2) *Stenotrophomonas* spp. (6)	Natural environments (water, soil, plants), and animal tissues. Include human pathogens	None	None	Efflux Inactivation Target modification Other	*Stenotrophomonas* 86.3% *Vibrio* 13.7%
17	*Desulfomicrobium* *Desulfomicrobiaceae* Delta- 7/1328	*Des. orale* (4) *Desulfomicrobium* spp*.* (3)	Natural environments (anoxic black mud, sewage, fresh- or brackish water) and gastrointestinal tract of animals	None	None	None	None
	*Desulfovibrio* *Desulfovibrionaceae* *Delta-* 7/1328	*Dv. desulfuricans* (1) *Dv. fairfieldensis* (1) *Dv. hydrothermalis* (1) *Desulfovibrio* spp. (4)	Natural environments (anoxic black mud, sewage, fresh- or brackish water) and gastrointestinal tract of animals	None	None	Inactivation	*Acinetobacter* 50.0% *Escherichia* 50.0%
	*Mannheimia* *Pasteurellaceae* *Gamma-* 7/1328	*Man. haemolytica* (2) *Man. varigena* (1) *Man. granulomatis* (1) *Man. ruminalis* (1) *Mannheimia* spp. (2)	Mucous membranes (upper respiratory tract of warm-blooded animals)	Others	*Actinobacillus* 100%	Efflux Inactivation Target modification Other	*Pasteurella* 33.9% *Escherichia* 13.3% *Pseudomonas* 12.5% *Mannheimia* 16.1% *Vibrio* 8.1% Others 16.1%
	*Shewanella* *Shewanellaceae* *Gamma-* 7/1328	*Sh. aquimarina* (1) *Sh. loihica* (1) *Sh. japonica* (1) *Sh. decolorationis* (1) *Shewanella* spp. (3)	Nutrient rich marine environments. Occasional clinical occurrence	Others	*Shigella* 100%	Efflux Inactivation Target modification	*Shewanella* 23.3% *Vibrio* 16.5% *Escherichia* 14.3% *Klebsiella* 14.3% *Salmonella* 9.8% *Pseudomonas* 6.8%
18	*Achromobacter* *Alcaligenaceae* *Beta-* 6/1328	*Ach. xylosoxidans* (4) *Achromobacter* spp*.* (2)	Natural environments (water, soil, plants), and animal tissues. Occasional clinical occurrence	Adherence Invasion Iron transport Transport Siderophore; Others	*Escherichia* 54.3% *Shigella* 32.6% *Salmonella* 8.7%	Efflux Inactivation Target modification Other	*Escherichia* 50.5% *Vibrio* 19.0% *Achromobacter* 13.3% *Pseudomonas* 7.6%
	*Ralstonia* *Burkholderiaceae* *Beta-* 6/1328	*Ralstonia* spp. (6)	Natural environments (water, soil, plants), and animal tissues. Occasional clinical occurrence	None	None	Inactivation Target modification	*Ralstonia* 97.0%
	*Rhizobium* *Rhizobiaceae* *Alpha-* 6/1328	*Rh. daejeonense* (1) *Rh. leguminosarum* (1) *Rh.sullae* (1) *Rh.loti* (1) Rhizobium sp. (2)	Plants (symbiotic nitrogen fixation)	Adherence Motility	*Campylobacter* 100%	Efflux Inactivation	*Agrobacterium* 69.2% *Salmonella* 7.7% Others 7.7%
19	*Bordetella* *Alcaligenaceae* *Beta-* 5/1328	*Bor. pertusis* (2) *Bor. petri* (1) *Bor. parapertussis* (1) *Bordetella* sp. (1)	Natural environments (water, soil, plants), and animal tissues. Include pathogens	Adherence Motility Endotoxin Host response evasion Others	*Bordetella* 100%	Efflux Inactivation Target modification Other	*Bordetella* 52.7% *Vibrio* 39.2%
	*Brevundimonas* *Caulobacteraceae* *Alpha-* *5/1328*	*Br. Diminuta* (2) *Brevundimonas* spp. (3)	Natural environments, (water, soil, plants), and animal tissues. Include pathogens	Adherence Motility	*Pseudomonas 100%*	Target modification	*Acinetobacter 40.0%* *Vibrio 60.0%*
	*Methylobacterium* *Methylobacteriaceae* *Alpha-* 5/1328	*Met. fujisawaense* (1) *Methylobacterium* spp. (4)	Natural environments (water, poor in nutrients and/or stress conditions	None	None	None	None
	*Pantoea* *Erwiniaceae* *Gamma-* 5/1328	*Pan. agglomerans* (1) *Pantoea* spp. 4/5	Natural environments (water, soil, plants), and animal tissues. Occasional clinical occurrence	Others	*Shigella* 43.2% *Salmonella* 35.4% *Escherichia* 21.4%	Efflux Inactivation Target modification Other	*Klebsiella* 40.3% *Shigella* 22.4% *Vibrio* 14.9% *Acinetobacter* 7.5% Others 6.0%
	*Proteus* *Morganellaceae* *Gamma-* 5/1328	*Pr. mirabilis* (2) *Proteus* spp. (3)	Gastrointestinal tract of warm-blooded animals. Also in natural environments (water, soil, plants)	Secretion system Others	*Escherichia* 55.6% *Vibrio* 22.2% *Shigella* 11.1% *Salmonella* 5.6% *Yersinia* 5.6%	Efflux Inactivation Target modification Reduced permeability Other	*Escherichia* 36.0% *Vibrio* 15.9% *Acinetobacter* 13.7% *Klebsiella* 10.1% *Salmonella* 8.9%
	*Psychrobacter* *Moraxellaceae* *Gamma-* 5/1328	*Psy. celer* (1) *Psychrobacter* spp. (4)	Natural environments (sea, ice), and animal tissues (skin, gills)	Adherence Motility Iron transport Transport Others	*Neisseria* 100%	Inactivation Target modification	*Escherichia* 41.2% *Vibrio* 23.5% *Psychrobacter* 17.6% *Acinetobacter* 5.9% *Neisseria* 5.9% *Oligella* 5.9%

^1^Antibiotic resistance and virulence determinants were identified using the criteria of 100% of subject and query sequence coverage and 100% of sequence identity—only generic functions are indicated

^2^Other taxa sharing a genetic element with 100% identical amino-acid sequence (only taxa representing more than 5% are indicated)

## The oral cavity as primary or transient *Pseudomonadota* habitat

To avoid situations of misidentification or of sporadic episodes of occurrence, the further discussion is focused at genus level identifications and on situations where consistent reports were available, i.e. genera that were reported more than five times. This procedure resulted in a list of 40 genera of the classes *Gammaproteobacteria* (*n* = 20), *Betaproteobacteria* (*n* = 11), *Epsilonproteobacteria* (*n* = 2), *Deltaproteobacteria* (*n* = 3), and *Alphaproteobacteria* (*n* = 4). For sake of simplicity, the 40 genera were ranked according to their frequency of occurrence, resulting in 19 categories designated from Top1 to Top19 (Table S3c, Table 1). In addition, the ecology and habitats, clinically relevant features (virulence and antibiotic resistance), and hypothetical horizontal gene transfer, suggested by the occurrence of identical genetic elements in other bacterial groups, are summarized in the five right-hand columns of Table 1. The association of members of almost all these genera (*n* = 36) to infectious disease has been demonstrated (Table 2), suggesting that specific host conditions may facilitate the opportunistic character of these bacteria.

**Table 2:** Examples of infectious diseases associated with *Pseudomonadota* reported in the oral cavity. Source: https://www.patricbrc.org (accessed at May 7^th^ 2022) and examples of additional references: (1) Pathak et al. 2021; (2) Cross et al. 2018; (3) Shrestha et al. 2021; (4) Calheiros Cruz et al. 2022; (5) Chi et al. 2004; (6) Nseir et al. 2011; (7) Hagiya et al. 2018; (8) Danger et al. 2022; (9) Zhou et al. 2021; (10) Chen et al. 2011

Infectious disease category	*Pseudomonadota* genera
Oral	*Aggregatibacter* *Campylobacter* (1) *Desulfovibrio* *Desulfobulbus* (2) *Eikenella*
Respiratory tract (bronchitis, pneumonia, meningitis, etc.)	*Achromobacter* *Actinobacillus* *Bordetella* *Burkholderia* *Haemophilus* *Klebsiella* *Mannheimia* *Neisseria* *Pantoea* (3) *Pseudomonas* *Psychrobacter* *Serratia* *Stenotrophomonas*
Gastrointestinal tract	*Aeromonas* *Campylobacter* *Escherichia* *Helicobacter* *Lautropia* (4) *Proteus*
Blood (sepsis, bacteremia)	*Acinetobacter* *Aeromonas* *Brevundimonas* (5) *Citrobacter* *Comamonas* (6) *Desulfovibrio* (7) *Escherichia* *Herbaspirillum* *Kingella* *Methylobacterium* *Moraxella* (8) *Neisseria* *Pantoea* *Pasteurella* *Proteus* *Ralstonia* (9) *Serratia* *Stenotrophomonas*
Other (arthritis, osteomyelitis, endocarditis, cellulitis, encephalitis, skin, etc.)	*Cardiobacterium* (10) *Citrobacter* *Enterobacter* *Haemophilus* *Kingella* *Neisseria* *Pasteurella* *Proteus* *Shewanella*

The assessment of the information compiled in Table 1 highlights three profiles—bacteria mainly associated with humans and other animals, environmental bacteria, and ubiquitous bacteria. Bacteria mainly associated with humans and other animals were represented mainly in the Top1–6 groups, composed of genera whose major habitat includes mucosa of oral cavity and sometimes also of the gastrointestinal and/or genital tract. Environmental bacteria were found in the Top7–19 groups, which include also genera associated with animals and humans (e.g. Top8, 13, some Top16, 17; Table 1) and others, whose natural environment (water, soils, plants) is the primary habitat. Genera such as *Rhizobium*, *Herbaspirillum*, *Shewanella*, or *Methylobacterium* are good examples of bacteria that typically thrive in the natural environment, and which presence in the oral cavity may be explained based on diet, lifestyle, or familiar context, with unknown potential clinical relevance (Table 1) (Hisham Altayb et al. 2022; Nasidze et al. 2011). Other groups, typically of environmental nature, like the strict anaerobic sulphate-reducing *Deltaproteobacteria*, such as members of the genera *Desulfovibrio*, *Desulfomicrobium*, and *Desulfobulbus*, have been reported in the oral microbiota of healthy people (Deo and Deshmukh 2019), although may be also associated with pathologies such as periodontal disease, dental plaques, or gastrointestinal inflammation (Colombo et al. 2009; Khor et al. 2021). The group of ubiquitous bacteria, with a wide distribution that span from pristine to heavily contaminated environments, and also the human and animal body, are those of major concern in the environment-human oral cavity interface. These bacterial genera, such as *Pseudomonas*, *Escherichia*, *Citrobacter*, *Klebsiella*, or *Burkholderia*, were in the groups Top7, 9, 11, 12, 16 (Table 1), include opportunistic pathogens, and frequently harbour acquired antibiotic resistance. The presence of these groups in the oral cavity may be associated with poor hygiene conditions, oral dysbiosis, deficient host defences, systemic diseases, or other factors; for example, chronic nail-biting habit and chronic kidney disease promote the oral carriage of *Enterobacteriaceae* (Baydaş et al. 2007; Costa et al. 2021). The ecology and genome plasticity of these ubiquitous bacteria may also explain the diverse array of antibiotic resistance and virulence mechanisms that characterize these groups (Table 1). The facts that members of these genera can thrive in soil, water, and plants, where human and animal excreta can be also present, and have typically highly dynamic genomes, suggest that these bacteria can serve as vectors of clinically relevant features (e.g. antibiotic resistance, virulence) from the environment to humans (Sanz-García et al. 2021; Wang et al. 2022a, 2022b). Indeed, the summary provided in Table 1 shows that bacteria of some of these genera hold a broad set of antibiotic resistance genes, generically included in the categories efflux, inactivation, target modification, reduced permeability, among others, that are shared by other *Pseudomonadota*. Also, virulence genes, related with factors such as adherence, invasion, transport, iron transport, secretion systems, motility/chemotaxis, toxins, host response evasion, among others are reported in bacteria of these genera. Virulence and antibiotic resistance determinants may contribute to enhance the capacity for colonizing, which can also be favoured by charity processes among the microbial community members, e.g. through extracellular antibiotic degradation. In some cases, identical gene sequences (100% sequence identity and coverage) were found in other taxa, suggesting the potential for dynamic horizontal gene transfer. Remarkably, bacteria of genera, such as *Achromobacter*, *Acinetobacter*, *Aeromonas*, *Bordetella*, *Burkholderia*, *Campylobacter*, *Citrobacter*, *Comamonas*, *Enterobacter*, *Escherichia*, *Haemophilus*, *Klebsiella*, *Pantoea*, *Pasteurella*, *Proteus*, *Serratia*, and *Shewanella*, harbour antibiotic resistance and/or virulence genes that are 100% identical to others reported in other taxa, whose habitats include the transition between animals, humans, soil, water, and plants. Although horizontal gene transfer is probably rare in the oral cavity (Tierney et al. 2019), the colonization by bacteria with a rich accessory genome comprised by antibiotic resistance or virulence genes, acquired somewhere else, is of concern, mainly in elderly, immune-depressed, and individuals with oral dysbiosis (Radaic and Kapila 2021). It is suggested that some *Pseudomonadota* with opportunistic pathogenic character and able to harbour acquired genes can reach the oral cavity and, eventually, can colonize it as well as the gut or other body habitats and ultimately cause disease. Moreover, it is noteworthy that some of these bacteria are probably able to cross the distinct One Health compartments and may represent important vectors of transmission of antibiotic resistance between the natural environment and the humans (Osterhaus et al. 2020). For some genera, specifically *Aggregatibacter*, *Cardiobacterium*, *Desulfobulbus*, *Desulfomicrobium*, *Desulfovibrio*, *Eikenella*, *Herbaspirillum*, *Lautropia*, *Methylobacterium*, *Ralstonia*, and *Simonsiella*, the data available on virulence or antibiotic resistance was scant. Although most of these genera have been associated with the oral cavity and human body and reported to thrive in natural environments with low anthropogenic impacts, further information about their genome plasticity and physiology may be relevant to better understand their role in the oral cavity.

## *Pseudomonadota*: from the oral cavity to the gut microbiome

An interconnection between the oral and gut microbiomes has been demonstrated in the literature (Khor et al. 2021; Kitamoto et al. 2020; Kitamoto and Kamada 2022). According to Kitamoto et al. (2020), bacteria can be transmitted from the oral cavity to the gut through hematogenous or enteral routes. In the hematogenous route, the bacteria have access to a systemic circulation through oral mechanical injuries (induced by, e.g. hard mastication, brushing, orthodontics, extractions, or unhealthy periodontium) with the subsequent gut colonization. In the enteral route, bacteria migrate through the gastrointestinal tract till colonizing the intestine. It is generally assumed that only part of the swallowed oral bacteria reaches and colonizes the healthy gut due to the gastric acidity. Indeed, the gut-resident- and oral microbiota are represented by distinct bacterial groups identified based on 16S rRNA gene sequence amplicon analysis (Wang et al. 2022b). Comparatively, *Pseudomonadota* are present at much lower proportions in the gut than in the oral cavity (< 5% vs. > 30%) (Wang et al. 2022b). However, when members of the oral bacterial community reach the gut, they may induce a considerably change in the gut microbiome composition, with systemic repercussions. For example, Nakajima et al. (2015) showed that the oral administration to mice of *Porphyromonas gingivalis* (phylum *Bacteroidota*) led to a decrease of *Bacillota* in the gut, while increased serum endotoxin levels were observed, suggesting the impairment of the barrier leading to the dissemination of enteric bacteria into the liver.

The search for closely related *Pseudomonadota* taxa in the oral cavity and of human gut suggested evidence for this nexus. The sequence identity between the 16S rRNA gene of the type strain of species reported in the oral cavity and human gut microbiome revealed values ranging from 78.1 to 98.6% (Table S4). Identity values above 97%, a threshold below which it is assumed that two organisms belong to different species (Stackebrandt and Goebel 1994), were observed for the species *Enterobacter cloacae*, *Enterobacter sakazakii* (valid name *Kosakonia sacchari*), *Enterobacter hormaechei*, *Klebsilela pneumoniae*, *Citrobacter koseri*, *Citrobacter amalonaticus*, and *Desulfovibrio desulfuricans*. In addition, although with lower sequence identity values (91.2–90.4%), significant alignments (e-values ranging from e^−87^ to e^−179^) were observed for species of the genera *Neisseria*, *Haemophilus*, *Campylobacter*, *Kingella*, *Pseudomonas*, *Eikenella*, *Actinobacillus*, *Acinetobacter*, *Moraxella*, and *Comamonas* (Table S4). The finding of significant sequence identities may be limited by multiple factors, specifically, the use of type strains 16S rRNA gene sequences, the incapacity to detect minor populations, as is the case of *Pseudomonadota*, due to low DNA sequencing depth of the human microbiomes, and the fact that the DNA sequences being compared do not belong to the same human microbiome (gut and oral). It must be noted that that nexus oral-gut is probably established based on minor populations. Indeed, Tierney et al. (2019) observed that although gut and oral microbiota genes are shared, that is observed to < 2.5% of the microbiome genes; i.e. considering 95% of sequence identity, only 549 610 genes were common to the oral (23 411 898 genes) and gut (21 704 828 genes) microbiomes. While the low depth of sequencing is a major limitation to reliably compare metagenomes, Tierney et al. (2019) argued that each individual holds a unique microbiome, which can be fingerprinted based on rare microbial strains. Clearly, some *Pseudomonadota* genera are particularly suitable candidates for such fingerprinting approach, for epidemiological and health condition evaluations. As suggested, even if in low numbers, some oral *Pseudomonadota* genera are likely to reach and colonize the human gut. Eventually, this situation may be favored or triggered by dysbiosis conditions, frequently associated with the increased relative abundance of *Pseudomonadota* in the oral or gut microbiome (Khor et al. 2021; Weiss and Hennet 2017). Indeed, the study of Atarashi et al. (2017) showed based on gnotobiotic models that *Klebsiella* spp. isolated from the salivary microbiota tend to colonize the gut when the intestinal microbiota is under dysbiosis, eliciting a severe gut inflammation by strongly inducing T helper 1 cells. These findings have been confirmed in subsequent studies (Kitamoto et al. 2020), who showed that the periodontitis-driven accumulation of *Klebsiella* spp. and *Enterobacter* spp. in the oral cavity might result in a consequent increase in the gut, possibly inducing colitis. Curiously, such effects were only observed in susceptible hosts, as that oral bacteria did not colonize the gastrointestinal tract of healthy animals (Kitamoto et al. 2020). These studies suggest that species of the phylum *Pseudomonadota*, despite constituting a small fraction of the oral microbiota, can colonize the gut, and contribute to maintain gut dysbiosis and chronic inflammation (Khor et al. 2021; Kitamoto and Kamada 2022).

## Final considerations

*Pseudomonadota* may include oral pathobionts, some of which constitute a reservoir of virulence and antibiotic resistance genes (Table 1, Table 2). In addition to the regularly found groups, others comprising the accessory, variable, or “non-core” microbiome (Deo and Deshmukh 2019) may be of interest, as indicators of specific health conditions or of an unbalanced microbiota. By default, the non-core microbiome is highly diverse and vast, probably influenced by the individual health conditions, dietary and lifestyle choices, hygiene practices, geography, and even ethnicity (Hisham Altayb et al. 2022; Wang et al. 2022b). In our review, we reported more than 250 bacterial genera that have not been listed as part of the core microbiota of the oral cavity, and which occurrence is probably minor in abundance and shaped by external variables. However, by highlighting a short list of 40 genera that were recurrently reported in the literature, this review suggests that non-core *Pseudomonadota* may be more diverse and frequent in the oral cavity than formerly believed, stressing the need of further investigation.

The increasing potential of anthropogenic bacteria to colonize, invade, and persist in environment-human interfaces, mainly when subjected to strong disinfection or antimicrobial actions, deserves attention (Becerra-Castro et al. 2016; Blaustein et al. 2021; Alexander Mahnert et al. 2019; Osterhaus et al. 2020). This review supports the hypothesis that humans exposed to food products and environments where increasingly anthropogenic microbiomes pullulate may have increased probability of acquiring antibiotic-resistant and virulent bacteria. These colonization events may only manifest under a host debilitation situation. The relationship between oral dysbiosis and oral (e.g. periodontal disease or dental caries) or systemic diseases (e.g. diabetes, cancer, endocarditis, systemic infections), where the unbalanced microbiome may be the trigger for the pathology, has been demonstrated (Albuquerque-Souza and Sahingur 2022; Al-Qadami et al. 2022; Khor et al. 2021). The interplay between the external factors and the colonization or proliferation in the oral cavity of some bacterial groups may be the key to prevent and control some pathologies. In addition, this is a crucial interface to better understand the environment-humans continuum that is implicitly assumed by the One Health concept.

## Supplementary information

Below is the link to the electronic supplementary material.

Supplementary file1 (XLSX 126 KB)

## Data Availability

Information about the sources of data supporting the results are reported throughout the text, with indication of the respective websites and access date.
